# Darbepoetin alfa given every 1 or 2 weeks alleviates anaemia associated with cancer chemotherapy

**DOI:** 10.1038/sj.bjc.6600465

**Published:** 2002-08-01

**Authors:** J A Glaspy, J S Jadeja, G Justice, J Kessler, D Richards, L Schwartzberg, N S Tchekmedyian, S Armstrong, J O'Byrne, G Rossi, A B Colowick

**Affiliations:** 200 UCLA Medical Plaza, Suite 202, Los Angeles, California, CA 90095-6956, USA; Hematology-Oncology Associates of Jacksonville, 5742 Booth Road, Jacksonville, Florida, USA; Pacific Coast Hematology Oncology Medical Group, 11190 Warner Avenue, Suite 300, Fountain Valley, California, USA; Virginia Oncology Associates, 895 Middle Ground Boulevard, Newport News, Virginia, USA; Tyler Cancer Center, 910 E Houston, Tyler, Texas, USA; The West Clinic, 100 N Humphries Blvd, Suite 100, Memphis, Tennessee, USA; Pacific Shores Medical Group, 1043 Elm Avenue, Suite 104, Long Beach, California, USA; Amgen Inc., One Amgen Center Drive, Thousand Oaks, California, CA 91320, USA

**Keywords:** anaemia, chronic disease, erythropoietin, neoplasms

## Abstract

In part A of this study, patients were randomised to cohorts receiving darbepoetin alfa at doses of 0.5 to 8.0 m.c.g. kg^−1^ wk^−1^ or to a control group receiving epoetin alfa at an initial dose of 150 U kg^−1^ three times weekly. In part B, the cohorts were darbepoetin alfa 3.0 to 9.0 m.c.g. kg^−1^ every 2 weeks or epoetin alfa, initial dose 40 000 U wk^−1^. Safety was assessed by adverse events, changes in blood pressure, and formation of antibodies to darbepoetin alfa. Efficacy was assessed by several haematologic endpoints, including change in haemoglobin from baseline. The adverse event profile of darbepoetin alfa was similar to that of epoetin alfa. No relationship between the rapidity of haemoglobin response and any adverse event was observed. No antibodies to darbepoetin alfa were detected. Higher doses of darbepoetin alfa increased the proportion of patients with a haemoglobin response and decreased the median time to response. The overall dose of darbepoetin alfa required to produce a mean increase in haemoglobin does not increase when the dosing interval is increased from 1 to 2 weeks. Therapy with darbepoetin alfa is safe and effective in producing a dose-related increase in haemoglobin levels in patients with cancer receiving chemotherapy.

*British Journal of Cancer* (2002) **87**, 268–276. doi:10.1038/sj.bjc.6600465 www.bjcancer.com

© 2002 Cancer Research UK

## 

Patients with cancer are frequently anaemic, with contributing factors including chemotherapy and the anaemia of chronic disease, with endogenous erythropoietin deficiency, relative erythropoietin resistance, and shortened red cell survival (Miller *et al*, 1990). It has been recognised that, even in the setting of mild and moderate degrees of anaemia, anaemia is an important contributing factor to the fatigue experienced by cancer patients (Vogelzang *et al*, 1997; Glaspy *et al*, 1997; Demetri *et al*, 1998; Gabrilove *et al*, 2001).

Recombinant human erythropoietin (rHuEPO) is effective for treating anaemia in patients receiving chemotherapy. Randomised trials demonstrated that increasing haemoglobin concentrations were associated with approximately a 50% reduction in administered transfusions (Cascinu *et al*, 1994; Henry and Abels, 1994). Large, open-label studies suggest that therapy with rHuEPO is associated with an improvement in energy level, ability to do daily activities, and overall quality of life (Demetri *et al*, 1998; Glaspy *et al*, 1997; Gabrilove *et al*, 2001). This improvement in the quality of life and fatigue level associated with rHuEPO treatment has been confirmed in a randomised, placebo-controlled clinical trial (Littlewood *et al*, 2001). The recognition that anaemia is an important factor in the quality of life of cancer patients has led to an increase in the use of rHuEPO in this setting (Gabrilove *et al*, 2001). Despite this success, the most effective dose and schedule of rHu-EPO to relieve fatigue as efficiently as possible have not been established.

Darbepoetin alfa is the recombinant product of a gene produced through site-directed mutagenesis of the erythropoietin gene that increases the glycosylation of the protein. Darbepoetin alfa binds to the erythropoietin receptor with the same fidelity and stimulates erythropoiesis by the same mechanism as endogenous erythropoietin and rHuEPO (Egrie *et al*, 1997). Despite darbepoetin alfa's reduced affinity for the erythropoietin receptor, it has increased potency due to its extended serum residence time (Egrie *et al*, 1997).

This increased *in vivo* potency has been confirmed in clinical trials in patients with renal failure (Macdougall *et al*, 1999). In this setting, darbepoetin alfa has a three-fold longer terminal half-life than rHuEPO (25.3 h *vs* 8.5 h) and can be administered less frequently with the same efficacy as rHuEPO.

In the cancer setting, the dosing recommendation for rHuEPO in the package insert (US) is 150 U kg^−1^ administered three times weekly, or approximately 10 000 U three times weekly. A large, phase 4 study has shown that increasing the label-recommended weekly dose by 33% to 40 000 U wk^−1^ administered as a single injection produces a haematologic response after 16 weeks of therapy in 49% of patients (Gabrilove *et al*, 2001). The response rate increased to 68% when the dose for patients with an inadequate response after 4 weeks was increased to 60 000 U wk^−1^. These response rates appear to be comparable to three times weekly dosing schedules using the lower overall rHuEPO doses (Demetri *et al*, 1998).

Clinical trials of darbepoetin alfa during cancer chemotherapy aimed at fully elucidating the dose–response relationships and optimising scheduling are underway. Initial data demonstrated that darbepoetin alfa can be administered as infrequently as once every 3 weeks (Kotasek *et al*, 2000). We report the results of a large, randomised, active-controlled, dose-finding study of darbepoetin alfa given subcutaneously every 1 or 2 weeks during cancer chemotherapy. Preliminary safety and efficacy results of the first weekly dose cohorts have been previously reported (Glaspy *et al*, 2001).

The purpose of this study was to assess the safety of darbepoetin alfa in patients receiving cancer chemotherapy, to assess the feasibility of administering darbepoetin alfa every week and every 2 weeks, and to characterise the dose–response relationships for darbepoetin alfa when given every week or every 2 weeks.

## PATIENTS AND METHODS

### Patients

The protocol was approved by the institutional review boards of participating centres; all patients gave written informed consent before any study-related procedures were done. Eligible patients were at least 18 years of age, had solid tumours, and were scheduled to receive cyclic chemotherapy for at least 12 weeks after enrolment. Patients were required to be anaemic (haemoglobin ⩽11.0 g dl^−1^) and to have an Eastern Cooperative Oncology Group performance status of 0 to 2 and adequate renal function (serum creatinine <2 mg dl^−1^). Patients with anaemia due to iron, folate, or B_12_ deficiency, haemolysis, bleeding, or active infection, and those with transferrin saturation <15% or serum ferritin concentration <10 ng ml^−1^, were excluded. Patients were excluded if they had received more than two red blood cell transfusions within 4 weeks or any red cell transfusions within 16 days of randomisation, had received epoetin alfa therapy within 8 weeks, or had known central nervous system metastases.

### Study drugs

Patients were randomised to receive darbepoetin alfa (ARANESP™, Amgen Inc., Thousand Oaks, CA, USA) or epoetin alfa (Procrit®, Ortho Biotech, Raritan, NJ, USA), the latter used in a fashion consistent with either the US package insert (part A) or clinical practice (part B), to provide a safety comparator for darbepoetin alfa in a patient population in which a high incidence of adverse events due to chemotherapy or malignancy was expected. In keeping with other studies with erythropoietic agents, a wide variety of chemotherapies, regimens, and schedules were used in the study.

### Study design

**Figure 1:**
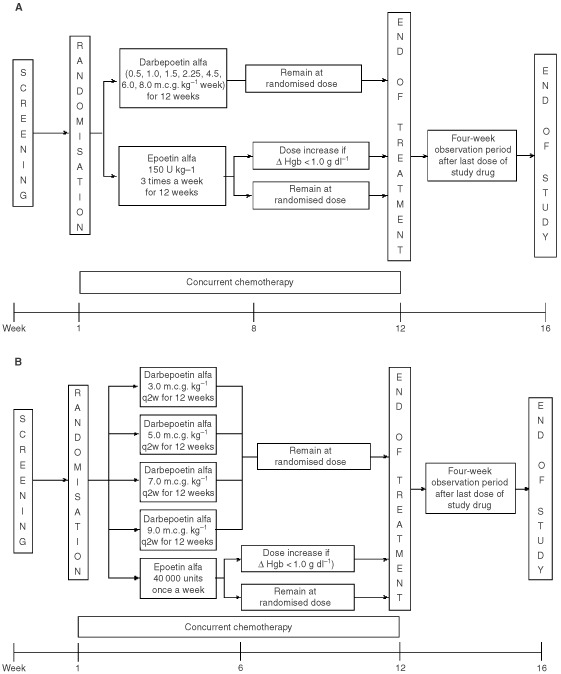
Study design and treatment schema. See text for information concerning dose adjustments for patients receiving epoetin alfa. In part A (**A**), darbepoetin alfa was administered once every week; in part B (**B**), it was administered once every 2 weeks.

This was a phase 1/2, multicentre, randomised, active-controlled, open-label study in which anaemic patients with solid tumours who were receiving multicycle chemotherapy received 12 weeks of subcutaneous therapy with darbepoetin alfa or epoetin alfa (Figure 1

). Part A was a sequential dose-finding study in which patients were randomised in a 4 : 1 ratio to receive either once-weekly doses of darbepoetin alfa (0.5, 1.0, 1.5, 2.25, 4.5, 6.0 or 8.0 m.c.g. kg^−1^) or epoetin alfa 150 U kg^−1^ three times weekly. In part A, dose-escalation decisions were determined by a data monitoring committee, based on specific safety criteria being satisfied. The exact size of each cohort was not prespecified, although a target size of 30 patients for each dose cohort was defined. Because of recommendations made by the data monitoring committee, additional patients were enrolled at some dose cohorts and three additional dose cohorts were included. Consequently, some variation in the actual size of the dose cohort exists. Part B was a parallel dose-finding and dose-schedule study in which patients were randomised in a 1 : 1 : 1 : 1 ratio to receive one dose of darbepoetin alfa (3.0, 5.0, 7.0 or 9.0 m.c.g. kg^−1^) once every 2 weeks or epoetin alfa 40 000 U wk^−1^. The randomisation allowed for approximately 30 patients to receive study drug per dose group. Study drug was administered on the first day of chemotherapy.

The doses of darbepoetin alfa were not increased to facilitate characterisation of the dose-response relationship. The study protocol allowed the dose of epoetin alfa to be increased for patients in whom an inadequate response (i.e., <1.0 g dl^−1^ increase in haemoglobin) was observed. In part A, the dose could be increased to 300 U kg^−1^ three times weekly at week 8. In part B, the dose could be increased to 60 000 U wk^−1^ at week 6. Because dose increases were allowed with epoetin alfa, the ability to directly compare the efficacy of an individual dose of darbepoetin alfa with epoetin alfa is confounded, although some descriptive comparisons can be made.

### Safety endpoints

The primary objective of this study was to assess the safety of darbepoetin alfa during cancer chemotherapy. All reported adverse events were grouped according to body system affected by preferred term according to a modified World Health Organisation adverse reaction term (WHOART) dictionary. In addition, changes in blood pressure, changes in haemoglobin concentrations, and the formation of antibodies to darbepoetin alfa were examined.

### Efficacy endpoints

The efficacy of darbepoetin alfa was assessed utilizing several endpoints related to haemoglobin concentration including haemoglobin response (⩾2.0 g dl^−1^ increase over baseline in the absence of a red cell transfusion in the preceding 28 days); time to haemoglobin response; change in haemoglobin from baseline at week 4 and week 13; and haematopoietic response (haemoglobin value >12.0 g dl^−1^ or a ⩾2.0 g dl^−1^ increase in haemoglobin over baseline value in the absence of a red cell transfusion in the past 28 days) (Gabrilove *et al*, 2001). An exploratory analysis was done to compare the efficacy of a similar weekly exposure to darbepoetin alfa when administration was every week or every 2 weeks.

The effect of changes in haemoglobin concentration on fatigue was studied using the Functional Assessment of Cancer Therapy–Fatigue (FACT-F) scale.

### Statistical analysis

Analyses were conducted using an intent-to-treat set that included all patients who received at least one dose of study drug. Baseline demographic and clinical characteristics were summarised by the mean for continuous measures and number for categorical measures. Changes in haemoglobin endpoints were summarised as a mean with 95% confidence limits. Time to haemoglobin response was summarised by Kaplan–Meier curves. The proportion of patients achieving haematopoietic response was estimated by subtracting the Kaplan–Meier estimate of the survivor function at the time of the last observed haematopoietic response from 1, with 95% confidence limits calculated by using Greenwood's estimate of the variance. FACT-F scale scores were summarised using medians with 95% confidence limits for all darbepoetin alfa and all epoetin alfa patients combined for the following haemoglobin categories: <0 g dl^−1^, 0 to <1.0 g dl^−1^ 1 to <2.0 g dl^−1^, 2.0 to <3.0 g dl^−1^, and ⩾3.0 g dl^−1^.

## RESULTS

### Patient demographics and disposition

At least one dose of study drug was received by 269 patients (216 darbepoetin alfa, 53 epoetin alfa) in part A and 160 patients (128 darbepoetin alfa, 32 epoetin alfa) in part B. Baseline characteristics of patients were well balanced (Table 1

**Table 1:**
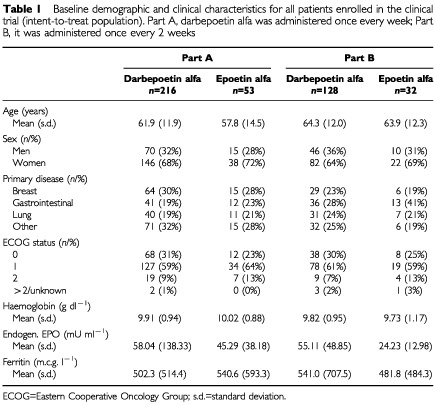
Baseline demographic and clinical characteristics for all patients enrolled in the clinical trial (intent-to-treat population). Part A, darbepoetin alfa was administered once every week; Part B, it was administered once every 2 weeks

). The proportion of patients completing 3 months of protocol treatment was similar for darbepoetin alfa and epoetin alfa (62 *vs* 57%, and 58 *vs* 63%, for darbepoetin alfa *vs* epoetin alfa in parts A and B, respectively). The reasons for early discontinuation of study drug treatment were similar for darbepoetin alfa and epoetin alfa patients and included death (5 *vs* 8%), disease progression (3 *vs* 2%), consent withdrawn (7 *vs* 9%), and chemotherapy discontinued or delayed (8 *vs* 9%), respectively. On average, the darbepoetin alfa cohorts received the intended dose with the exception of the 2.25 m.c.g. kg^−1^ wk^−1^ cohort in part A, in which the mean dose received was 2.05 m.c.g. kg^−1^ wk^−1^ due to protocol-specified dose reductions for rapid haemoglobin increases. The protocol was subsequently amended to remove these dose reduction criteria.

### Safety

**Figure 2:**
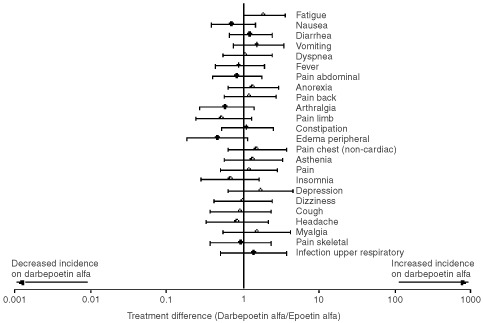
Comparison of adverse events that occurred with ⩾10% incidence in patients receiving darbepoetin alfa and epoetin alfa. Bars represent 95% confidence limits of the odds ratio.

The most frequently reported adverse events were those expected in a population of cancer chemotherapy patients and occurred at a similar frequency within the darbepoetin alfa and epoetin alfa groups (Figure 2

). For all events other than fatigue, the 95% confidence limits of the odds ratio include 1, suggesting no difference between darbepoetin alfa and epoetin alfa. No apparent relationship was noted between an increasing rate of any adverse event and the dose of darbepoetin alfa. The rates of serious adverse events and adverse events of grade 3 or greater were similar in patients receiving darbepoetin alfa and in those receiving epoetin alfa.

No relationship was noted between the changes in haemoglobin and changes in blood pressure or between the rate of change in haemoglobin and any specific adverse event, including cardiovascular and thrombotic events.

The effect of discontinuing darbepoetin alfa on the trajectory of the rise in haemoglobin was assessed in patients who met prespecified haemoglobin thresholds (14.0 g dl^−1^ for women and 15.0 g dl^−1^ for men). A plateau of the haemoglobin concentration was observed within 1 to 2 weeks, followed by a gradual decline until the study drug was reinstated per protocol when the patient's haemoglobin reached 13.0 g dl^−1^. Two women (one darbepoetin alfa and one epoetin alfa) exceeded the normal range of haemoglobin during the study without any clinical sequelae. Despite the longer half-life of darbepoetin alfa, the rate of decline was comparable to that of epoetin alfa and did not appear to be dose dependent.

Patients' sera were screened at regular intervals using a radioimmunoprecipitation assay to detect antibodies to darbepoetin alfa. No serum reactivity was observed within 1200 assays done on samples from 205 patients exposed to 1800 patient-weeks of darbepoetin alfa. In addition, no clinical sequelae suggestive of the formation of neutralising antibodies were observed.

### Efficacy endpoints

**Figure 3:**
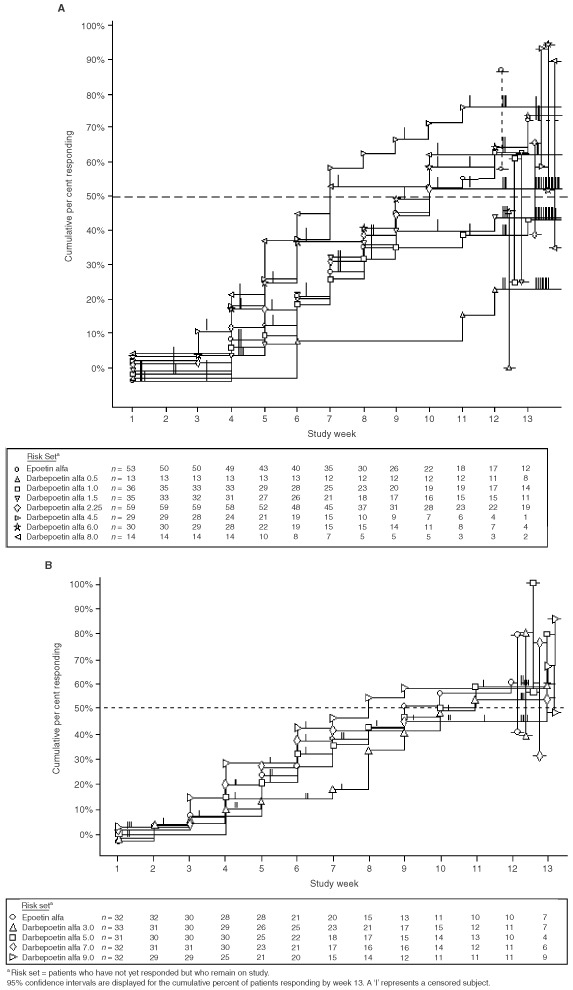
Cumulative proportion of patients achieving a haemoglobin response (defined as a ⩾2.0 g dl^−1^ increase over baseline). Patients who did not manifest a haemoglobin response to epoetin alfa had their dose increased at week 8 in part A and week 6 in part B. In part A (**A**), darbepoetin alfa was administered once every week; in part B (**B**), it was administered once every 2 weeks.

The cumulative proportion of patients achieving a haemoglobin response is shown in Figure 3

. A dose–response relationship was evident for patients receiving darbepoetin alfa once every week up to doses of 4.5 m.c.g. kg^−1^. Higher doses did not appreciably increase efficacy for a given dose cohort. The dose response was evident not only for the overall proportion of patients achieving a response but also for rapidity of the response. At 4.5 m.c.g. kg^−1^, the median time to response was 7 weeks (confidence limits, 6 to 10 weeks) compared with 10 weeks (confidence limits, 8 weeks to unestimable) for patients receiving 2.25 m.c.g. kg^−1^ or those receiving epoetin alfa. A dose response was not as evident for the doses administered once every 2 weeks, which all produced haematologic responses in more than 60% of the patients and which suggested a dose/time-to-response relationship.

**Figure 4:**
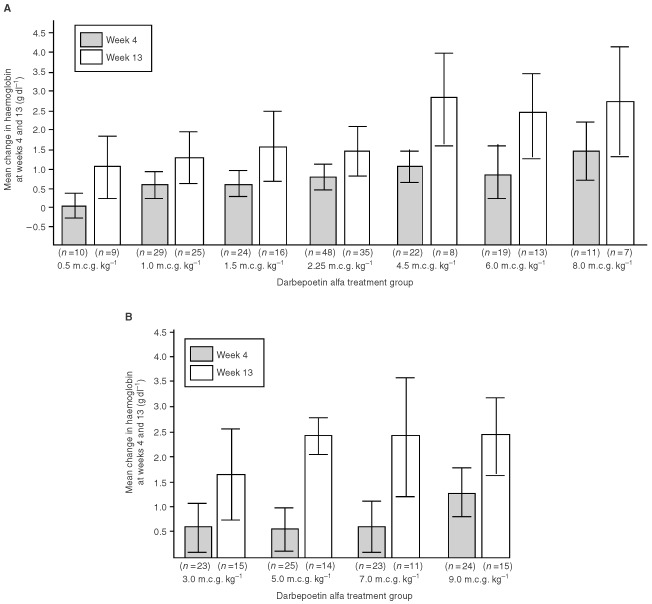
Mean change from baseline in haemoglobin at weeks 4 and 13. In part A, darbepoetin alfa was administered once every week; in part B, it was administered once every 2 weeks. Bars represent standard error of the mean.

The mean change in haemoglobin associated with the various doses and schedules of darbepoetin alfa was examined (Figure 4

). In part A, a trend towards greater increases in haemoglobin with higher doses of darbepoetin alfa was observed: the rise was 1.4 g dl^−1^ in the 0.5 m.c.g. kg^−1^ wk^−1^ cohort compared with a rise of 2.75 g dl^−1^ in the 8.0 m.c.g. kg^−1^ wk^−1^ cohort. Similarly, in part B, higher doses of darbepoetin alfa were generally associated with a greater change in haemoglobin compared with lower doses.

It is clinically relevant to consider earlier time points than week 13 (end of treatment) for assessing the impact of therapy on haemoglobin concentration. In part A, a trend towards greater increases in haemoglobin with higher doses of darbepoetin alfa was observed (Figure 4). In part B, patients receiving darbepoetin alfa doses of 3.0, 5.0 and 7.0 m.c.g. kg^−1^ every 2 weeks had similar mean increases in haemoglobin at week 4 (0.61, 0.58 and 0.61 g dl^−1^, respectively), whereas patients receiving darbepoetin alfa at 9.0 m.c.g. kg^−1^ every 2 weeks had a larger mean increase of 1.23 g dl^−1^.

**Figure 5:**
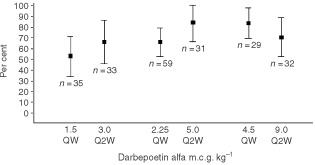
Plot of Kaplan–Meier proportion of patients with haematopoietic response during the treatment phase. Part A, darbepoetin alfa was administered once every week; Part B, darbepoetin alfa was administered once every 2 weeks. Bars represent 95% confidence limits.

We assessed the relative efficiency of the same overall dose of darbepoetin alfa when administration was weekly compared with every 2 weeks (Figure 5

). For each of the dosing couplets for which such a comparison is possible, the proportion of patients achieving a haematopoietic response was similar regardless of dosing interval. This finding suggests a linear relationship for dose requirements as the interval of dosing is increased from every week to every 2 weeks.

Of the 429 patients in the intent-to-treat analysis, fatigue score data were available for 408 (329 darbepoetin alfa, 79 epoetin alfa). Due to the small size of each dose cohort, the impact on fatigue, using changes in FACT-F scores, was analysed by change in haemoglobin category rather than by dose group/schedule of administration. The effect of a given change in haemoglobin concentration on FACT-F scores was similar between darbepoetin alfa- and epoetin alfa-treated patients (Table 2

**Table 2:**
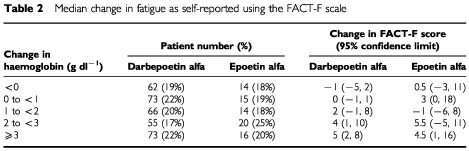
Median change in fatigue as self-reported using the FACT-F scale

).

## DISCUSSION

The recognition that fatigue is a major determinant of the functional status and quality of life of cancer patients and that mild degrees of anaemia contribute to this fatigue, coupled with the demonstration that increasing haemoglobin concentrations can reduce these fatigue levels, has led to an increase in the use of rHuEPO in this setting. Most studies have used three times weekly doses of 150 U kg^−1^, with a dose increase to 300 U kg^−1^ in non-responders (Cascinu *et al*, 1994; Henry and Abels, 1994). This schedule remains indicated for rHuEPO, although common practice in the US is to administer rHuEPO once a week. The dosing and scheduling of rHuEPO during cancer chemotherapy are increasingly important issues, as they affect the proportion of patients who benefit and the cost-utility of this therapy. It is important that new erythropoietic agents be developed with attention to optimised dose and schedule. This paper is the final report of a randomised, active-controlled, dose-finding trial of darbepoetin alfa therapy given every week or every 2 weeks during cancer chemotherapy.

The primary endpoint of this study was the safety of darbepoetin alfa. The adverse event profile observed with darbepoetin alfa was similar to that observed with epoetin alfa, dominated by those events expected in this patient population. Serious adverse events were frequent but were related to the cancer or the chemotherapy. Fatigue was reported as an adverse event more frequently in patients receiving darbepoetin alfa than in those receiving epoetin alfa. Because the darbepoetin group included patients treated at relatively low doses in whom a suboptimal haemoglobin response was observed, it is likely that the increased fatigue reported was related to excess anaemia. Using the validated FACT-F instrument, we observed that for a given improvement in haemoglobin, an equivalent decrease in fatigue is observed with both darbepoetin alfa and epoetin alfa.

In studies of patients with renal failure undergoing dialysis, rapid changes in haemoglobin associated with rHuEPO therapy may be associated with an increased incidence of hypertension and thrombosis of dialysis shunts. These phenomena are thought to be related to increasing red cell mass in the absence of normal-volume homeostasis. These complications have not been observed in the setting of cancer chemotherapy (Glaspy *et al*, 1997; Demetri *et al*, 1998; Gabrilove *et al*, 2001; Littlewood *et al*, 2001). In our study, no relationship between the rapidity of haemoglobin response and any adverse event was observed, consistent with the clinical experience with rHuEPO in cancer patients. Finally, although darbepoetin alfa is a longer-acting erythropoietic agent than epoetin alfa, only two patients who had haemoglobin >14 or 15 g dl^−1^ had haemoglobin concentrations that continued to increase to above normal range when darbepoetin alfa therapy was discontinued; no clinical sequelae were associated with these increases. Darbepoetin alfa appears to be safe and well tolerated in the setting of cancer chemotherapy.

Recently, a number of cases of pure red cell aplasia associated with antibodies to one type of rHuEPO (Eprex) have been reported in patients with chronic renal failure (Casadevall *et al*, 2002). These cases do not appear to be associated with other preparations of epoetin alfa (Epogen, Procrit). The neutralizing antibodies bound only to the protein moiety and not to the carbohydrate moiety of the rHuEPO and crossreacted with all commercially available recombinant erythropoietic products, including darbepoetin alfa. Darbepoetin alfa is a modified erythropoietin molecule with five amino acid substitutions and more sialic acid than native erythropoietin or rHuEPO. Because the amino-acid substitutions are at, or proximal to, the carbohydrate addition site, it is likely that these new epitopes are shielded from immune surveillance (Egrie and Browne, 2001). The type of carbohydrate moiety present on darbepoetin alfa (six chains) and rHuEPO (four chains) are identical. Darbepoetin alfa has been administered for up to 2 years without serum immunoreactivity being observed in patients with renal failure who were undergoing dialysis (Macdougall, 2001). In our study, no serum immunoreactivity was observed within 1200 assays done on samples from 205 patients exposed to darbepoetin alfa for 1800 patient-weeks. These observations are consistent with those in other studies of darbepoetin alfa administered to cancer patients (Kotasek *et al*, 2000; Heatherington *et al*, 2001; Smith *et al*, 2001). These clinical data are consistent with the preclinical prediction that anti-darbepoetin alfa antibody responses would not occur.

An objective of this study was to explore the dose-response relationship for darbepoetin alfa. Our data demonstrate that when darbepoetin alfa is administered every week, the proportion of patients who demonstrate a hematologic response increases as the dose is increased up to 4.5 m.c.g. kg^−1^ wk^−1^. At that dose, approximately 76% (95% confidence limit: 59 to 94%) of the patients met criteria for a haemoglobin response, and 80% (95% confidence limit: 70 to 98%) had a haematopoietic response (either normalised or responded). When darbepoetin alfa was administered every 2 weeks, a similar relationship between the proportion of responders and the dose administered was observed up to a dose of 9 m.c.g. kg^−1^ every 2 weeks. At that dose, 67% (95% confidence limit: 48 to 85%) of patients were responders, and 71% (95% confidence limit: 53 to 89%) either normalised or responded. Because intrapatient dose escalation was not permitted in this study for patients receiving darbepoetin alfa, these data do not speak to whether higher doses may allow for an individual patient to respond to darbepoetin alfa. Further studies are warranted in which patients who do not exhibit an early haemoglobin response are allowed to increase their dose of darbepoetin alfa to define the effect of dose escalation.

Similar trends towards increasing efficacy with increasing doses of darbepoetin alfa were observed for other efficacy endpoints, including the mean change in haemoglobin after 4 weeks and at the end of study (typically week 13). Importantly, a relationship between dose and rapidity of response is apparent, with higher doses of darbepoetin alfa achieving faster responses than lower doses or epoetin alfa. The median time to response for patients receiving 4.5 m.c.g. kg^−1^ wk^−1^ was 7 weeks (confidence limit: 6 to 10) compared with 10 weeks (confidence limit: 8 to unestimable) observed for patients receiving 2.25 m.c.g. kg^−1^ darbepoetin alfa every week or epoetin alfa 150 U kg^−1^ administered three times weekly, including the dose increase for nonresponding patients. This rapid response should translate into more rapid relief of fatigue and improvement in functional status.

Another observation is that the results achieved with darbepoetin alfa administered every week are similar to those achieved with twice the dose administered every 2 weeks. This finding may contrast with the results with epoetin alfa, where it is possible that a 33% increase in the total dose three times weekly is necessary for once-every-week dosing (Gabrilove *et al*, 2001). Darbepoetin alfa may permit more flexible scheduling of erythropoietic therapy without compromising cost-effectiveness.

This study has limitations. Because it was designed to be a dose-finding study, a direct comparison of the clinical effects of epoetin alfa and a given dose or schedule of darbepoetin alfa is not feasible. We administered darbepoetin alfa at the same maximum dose within each cohort without allowing for dose increases in those patients who did not respond early to therapy. Epoetin alfa dose escalations were permitted for patients in whom an inadequate early response was observed. Thus, any comparison of efficacy between the two drugs is likely to underestimate the full potential of a given starting dose of darbepoetin alfa. Second, at least with doses of up to 8 m.c.g. kg^−1^ wk^−1^, we have not eliminated the important clinical problem of non-responders to erythropoietic therapy. With the data available, we cannot determine the proportion of patients who would remain unresponsive if higher doses of darbepoetin alfa were used. Third, our entry criteria permitted participation by patients who may have had marginal or deficient iron reserves that may have compromised their response to epoetin alfa or darbepoetin alfa. Although these entry criteria were similar to those used in other studies of erythropoietic agents for cancer patients (Glaspy *et al*, 1997; Demetri *et al*, 1998; Gabrilove *et al*, 2001), it is possible that higher response rates or lower epoetin alfa or darbepoetin alfa doses would have been observed if more strict entry criteria had been used or if more aggressive iron supplementation had been mandated.

We conclude that darbepoetin alfa therapy is safe when administered to cancer patients receiving chemotherapy. An apparent relationship appears to exist between darbepoetin alfa dose and both the magnitude and rapidity of haematologic response. The overall dose of darbepoetin alfa required to produce a given mean increase in haemoglobin concentration does not increase when the dosing interval is increased from 1 to 2 weeks, allowing for greater flexibility when managing a patient's anaemia without adversely affecting the cost of therapy for the benefits of less-frequent dosing.
